# Does the availability of prior mammograms improve radiologists’ observer performance?—a scoping review

**DOI:** 10.1259/bjro.20230038

**Published:** 2023-10-18

**Authors:** Judith D. Akwo, Phuong Trieu, Sarah Lewis

**Affiliations:** 1 Medical Image Optimisation and Perception Group, Sydney School of Health Sciences, Faculty of Medicine and Health, The University of Sydney, Camperdown, Australia; 2 Orange Radiology, Laboratories, and Research Centre, Calabar, Nigeria

## Abstract

**Objective:**

The objective of this review was to examine the impact of previous mammogram availability on radiologists’ performance from screening populations and experimental studies.

**Materials and Methods:**

A search of the literature was conducted using five databases: MEDLINE, PubMed, Web of Science, ScienceDirect, and CINAHL as well as Google and reference lists of articles. Keywords were combined with “AND” or “OR” or “WITH” and included “prior mammograms, diagnostic performance, initial images, diagnostic efficacy, subsequent images, previous imaging, and radiologist’s performance”. Studies that assessed the impact of previous mammogram availability on radiologists’ performance were reviewed. The Standard for Reporting Diagnostic Accuracy guidelines was used to critically appraise individual sources of evidence.

**Results:**

A total of 15 articles were reviewed. The sample of mammogram cases used across these studies varied from 36 to 1,208,051. Prior mammograms did not affect sensitivity [with priors: 62–86% (mean = 73.3%); without priors: 69.4–87.4% (mean = 75.8%)] and cancer detection rate, but increased specificity [with priors: 72–96% (mean = 87.5%); without priors: 63–87% (mean = 80.5%)] and reduced false-positive rates [with priors: 3.7 to 36% (mean = 19.9%); without priors 13.3–49% (mean = 31.4%)], recall rates [with priors: 3.8–57% (mean = 26.6%); without priors: [4.9%–67.5% (mean = 37.9%)], and abnormal interpretation rate decreased by 4% with priors. Evidence for the associations between the availability of prior mammograms and positive-predictive value, area under the curve (AUC) from the receiver operating characteristic curve (ROC) and localisation ROC AUC, and positive-predictive value of recall is limited and unclear.

**Conclusion:**

Availability of prior mammograms reduces recall rates, false-positive rates, abnormal interpretation rates, and increases specificity without affecting sensitivity and cancer detection rate.

## Introduction

Breast cancer is identified as the second highest cause of cancer-related deaths in females across the world.^1,2^ The World Health Organisation’s (WHO) global estimate that 7.8 million women live with breast cancer.^3^ In 2020, there were 2.3 million new cases of breast cancer and 685,000 deaths from the disease.^3^ The incidence and deaths from breast cancer vary across the world,^2,4^ and in the last two decades, mortality from breast cancer has reduced considerably due to early detection primarily through organised population screening programmes and advances in treatment strategies.^2,5^ Current data show that advances in breast cancer screening and treatment have reduced breast cancer mortality by 20–40%.^5,6^ Full-field digital mammography (FFDM) is the standard imaging tool for breast cancer screening in Australia, the USA and many European countries, and has been partly credited with the success in improving survival rates from the disease.^2,5^ For mammographic images to be useful in the early detection of breast cancer, they must be accurately interpreted by trained radiologists and in some countries by breast physicians and advanced practice radiographers. However, heterogeneity in the appearance of the breast and breast cancer features, and anatomical noise due to tissue superposition make accurate interpretation of FFDM images challenging.^7^ These factors together with the inherent limitations of humans interpretating the images reduce the diagnostic performance of FFDM.^7,8^

Despite the introduction of digital breast tomosynthesis (DBT), ultrasound and MRI into the breast imaging pathway for screening and diagnosis, FFDM continues to be the frontline tool for breast cancer screening in many countries.^8,9^ In the last two decades, both technological and human-related strategies have been explored to improve the performance of FFDM in breast cancer screening.^8,10^ These technological strategies include computer-assisted software packages such as computer-aided detection (CAD), artificial intelligence (AI), and machine learning (ML) algorithms to flag suspicious areas in mammograms or provide diagnostic decision-support.^8,10^ Although these tools have shown promise for improving cancer detection, they are not widely used clinically, and the final diagnostic decision is still mostly made by radiologists. Therefore, strategies for improving radiologists’ performance have been the focus of many research studies.^8,11–13^ These strategies have included double independent reading of FFDM, pairing of radiologists with the technological algorithms, identification of radiologists’ characteristics associated with performance, and giving radiologists access to the previous images of screened females.^8,14,15^ These human-related strategies have been shown to impact differently on radiologist’s performances and understanding which of these factors improves performance is crucial to improving the outcomes of breast cancer screening programs.

The detection of breast cancer in mammograms of asymptomatic females is made based on the identification of abnormal changes displayed on these mammograms.^16^ With the digitisation of the screening process, it has become easier to retrieve and compare previous and current screening mammograms for abnormal breast changes. However, screening programs are also challenged by storage capacity due to the big data generated from current and previous screening rounds in the digital era and movement of females between screening services and public *vs* private clinic attendance for imaging. Consequently, several prospective and retrospective studies have examined the association between availability of previous mammograms and radiologists’ performance, but these studies have generated mixed outcomes.^17–22^ The reasons for these findings are not clear, and no review has been conducted to establish the relevance of prior mammograms to screening programs. Therefore, this review aims to examine the impact of previous mammograms on diagnostic performance in screening populations and experimental studies. Findings from the review should provide evidence for the relevant usability of prior imaging in clinical practice.

## Methods

### Eligibility criteria

Articles were considered eligible if they examined the impact of prior mammograms (FFDM, film-screen (FS) mammograms) on the accuracy of mammography interpretation and were published in the English language. We also included articles that used DBT in conjunction with mammography, in recognising that some screening and diagnostic imaging pathways have transitioned to the dual use of FFDM and DBT, or as DBT as an updated technology and FFDM was used for the prior cases. Articles were also considered eligible if they compared the performance of readers with and without prior images, had at least one reader and a reference standard for assessing reader performance. Articles that calculated at least one of the following metrics, sensitivity, specificity, recall rates (RRs), false-positive rates (FPRs), cancer detection rate (CDR), negative-predictive value (NPV), positive-predictive value (PPV), PPV of recall, abnormal interpretation rates (AIR), area under the curve from receiver operating characteristics (ROC AUC), Region-of-interest (ROI) figure of merit (FOM), jacknife alternative free response curve (JAFROC), location receiver operating characteristic curve (LROC), PPV of recall, and biopsy recommendation rate (BRR), were considered eligible. No restriction was placed on publication date.

### Information sources

A search of the literature was conducted using five databases: MEDLINE, PubMed, Web of Science, ScienceDirect, and CINAHL to identify articles that examined the impact of prior mammograms on radiologic image interpretation or diagnostic accuracy. Other information sources included Google, Google Scholar, and reference lists of published articles.

### Search strategy

The following keywords were used to search for articles and were combined with “AND” or “OR” or “WITH”: prior images AND diagnostic performance; initial images AND diagnostic efficacy; initial OR subsequent images OR previous imaging AND radiologist’s performance; prior radiographs AND reader performance; prior mammograms AND diagnostic efficacy; prior mammograms AND screening outcomes; previous imaging OR initial film; comparison WITH priors; prior breast imaging AND screening performance. A Google cross-search was conducted and reference lists of identified articles were also manually searched to identify additional articles.

### Study/source of evidence selection

After the articles were identified, their titles and abstracts were first screened by one reviewer. Full texts of articles that fulfilled the inclusion criteria were retrieved. A second reviewer independently reviewed the abstracts of these articles identified for relevance. Thereafter, a detailed assessment of the full text of all selected articles was performed against the inclusion criteria by two independent reviewers. Where there was a disagreement, this was resolved through discussion. Articles that did not fulfill these criteria were excluded as were reviews, case reports, and conference proceedings. The results of the search and evidence selection were then presented in a Preferred Reporting Items for Systematic Reviews and Meta-analyses extension for scoping review (PRISMA-ScR) flow diagram.^23^

### Data extraction/charting process

A data charting table was developed to guide data extraction from eligible articles. Each of the two reviewers, who were involved in the evidence selection process working independently, extracted the data from papers that fulfilled inclusion criteria based on this data charting table. The data extracted included study year, population of study/participants, study context and design, key findings relevant to the review question and adjustments. Any disagreements between the reviewers were also resolved via discussion, and when a consensus could not be reached, a third reviewer was contacted to act as an arbiter.

### Data items

Data items are summarised in Table 1 and included CDR, sensitivity, specificity, RR, ROC AUC, JAFROC, FPR, ROI FOM, LROC, PPV, AIR, and BRR.

**Table 1.** Data items included in the review

	Data items	Definition of data items
1	Cancer detection rate	The number of cancers detected per 1000 women screened.
2	Sensitivity	The rate of true-positive (cancer) cases that were correctly identified.
3	Specificity	The rate of true-negative (normal) cases that were correctly identified.
4	Recall rate	The rate of females recalled for follow-up imaging due to suspicious of cancer.
5	AUC from ROC	A measure of performance in correctly classifying normal and abnormal cases
6	JAFROC FOM	A measure of performance in localising a lesion and simultaneously rating the level of malignancy *vs* identifying correct normal cases
7	FPR	The number of negative cases that were incorrectly reported as positive.
8	ROI FOM	The empirical probability that a cancer containing ROI is rated higher than a normal ROI)
9	PPV	The proportions of positive results that are true positive
10	AIR	The number of abnormal findings that require additional follow-up or the number of mammograms with abnormal final interpretation.
11	Biopsy rate	The proportion of females had been recalled at screening and had a biopsy test.
12	LROC	A performance measure that quantifies readers ability to detect and locate an abnormality on the mammogram.

AIR, abnormal interpretation rate; AUC, area under the curve; FOM, figure of merit; FPR, false-positive rate; JAFROC, jacknife alternative free-response curve; LROC, location ROC; PPV, positive-predictive value; ROC, receiver operating characteristic; ROI, region-of-interest.

### Critical appraisal of individual sources of evidence

Critical appraisal was performed using the Standard for Reporting Diagnostic Accuracy (STARD) recommendations.^24^ Items assessed included research questions or study aims and methodological criteria such as participants, reference standard, blinding of readers, index text, flow and timing (interval between the index tests and the reference standard). Other items included methods for calculating measures of accuracy and quantify uncertainty, data collection, sampling, training and expertise of readers, characteristics of the population studied, description of disease distribution and severity, and reproducibility estimates.

## Results

### Selection of sources of evidence

**Figure 1.**
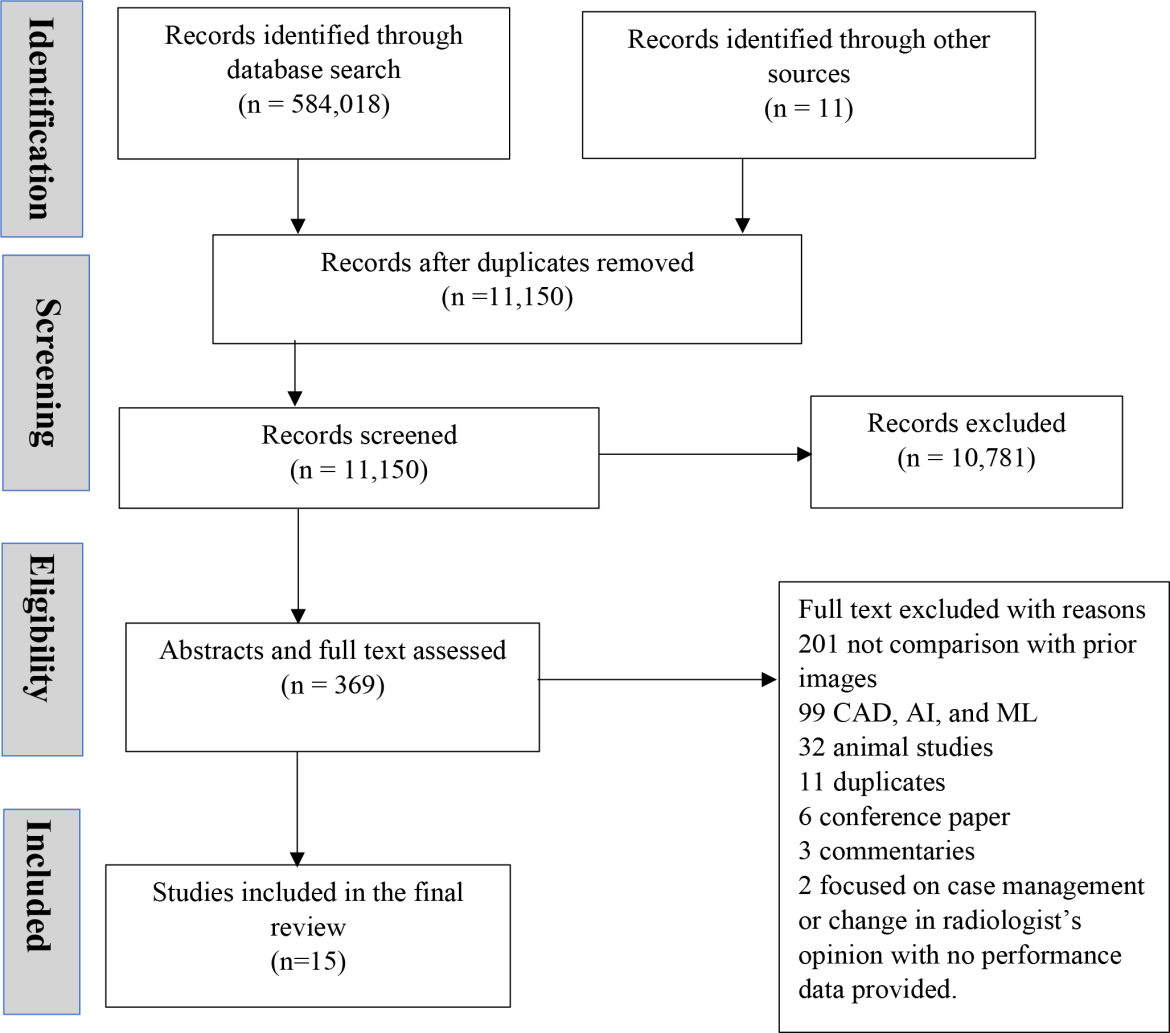
Prisma flow chart showing the selection of evidence. AI, artificial intelligence; CAD, computer-aided detection; ML, machine learning.

Database search yielded 584,018 articles and 11 articles were identified through other sources, including Google and reference list of published articles. After duplicates were removed, 11,150 articles were available for screening. Of these 11,150 articles screened, 10,781 were excluded because they were not mammography reader performance studies. Out of the remaining 369 articles selected for abstract and full-text review, 354 studies were excluded for the following reasons: (1) they did not compare reader performance with and without prior images (*n* = 201), were based on computer-aided detection such as AI or ML (*n* = 99) or were animal studies (*n* = 32). Duplicates (*n* = 11), conference papers (*n* = 6), commentaries or letters to the editor (*n* = 3), and studies that assessed change in case management or radiologist’s opinion but did not report any performance metric (*n* = 2) were excluded. A total of 15 articles fulfilled the eligibility criteria and were included in the review (Figure 1).

### Characteristics of sources of evidence

Table 1 summarises the characteristics of the studies identified. In terms of study location, there were 2 studies each conducted in the UK and Netherlands, 10 studies conducted in the USA, and 1 study each in Korea, Australia, and Denmark. 11 of these studies were observer performance studies examining test-sets^17,19–22,25–29^ while four were retrospective analyses of mammographic databases, with three examining screening data whilst one also included a cohort of diagnostic mammograms. Eight studies were either based on FS mammography or a combination of FS and FFDM; four studies used FFDM alone, and three studies used FFDM and DBT.^19,20,25^ The number of mammograms used in the studies reviewed ranged from 36 to 1,208,051 (Median: 160; Mean: 80,536.7). The sample of readers in 14 studies varied from 3 to 12, and 1 retrospective study evaluated test-set results of 612 radiologists.^30^ In these 15 studies, the index test (the test being evaluated against a reference standard) was independently interpreted in 13 studies, and 2 studies did not provide any information about the index test.^31,32^

In comparing performance with and without prior images, four studies used ROC AUC,^17,20,25,29^ four used RRs,^19,20,31,33^ seven used sensitivity and specificity,^21,22,25,28,30^ and four used CDR.^31,33,34^ Two studies examined diagnostic accuracy. Outcomes metrics such as PPV of recall, number of cancers detected, abnormal interpretation rate, biopsy recommendation rate, and biopsy yield were each assessed in one study. In addition, JAFROC FOM, LROC, false-positive recall, FPR, and ROI FOM were each assessed in one study (Table 2).

**Table 2.** A summary of the characteristics of the studies identified

Author/Year	Study location	Study design	Sample size	Participants/readers	Independent interpretation of index test	Outcomes/performance metrics			Key findings	Adjustments
						Outcome assessed	With priors	Without priors		
Callaway et al.1997^17^	UK	Observer performance	FS (*n* = 100): Normal: 88; Cancer:12	Radiologists (*n* = 8)	Yes	ROC AUC	0.78*^a^	0.81*^a^	Prior mammograms did not improve diagnostic accuracy	None
Hakim et al. 2014^19^	USA	Observer performance	DM+DBT (*n* = 36)	Radiologists (*n* = 8)	Yes	RRs	27%	64%	Prior mammograms reduced RRs	None
Kim et al. 2017^25^	Korea	Observer performance	DM+DBT *n* = 116 Cancers: 24	Radiologists (*n* = 3)	Yes	AUC	81%	82%	Adding prior mammograms did not significantly affect the AUC of DM +DBT; Prior mammograms significantly improved the specificity and PPV of DM +DBT; prior mammogram did not affect sensitivity of DM+DBT	None
						Sensitivity	69.4%	69.4%		
						Specificity	93.8%	85.9%		
						PPV	74.6%	56.2%		
Hayward et al. 2016^33^	USA	Retrospective	Prior = FS Current = DM *n* = 6,288	Radiologists. number not reported	Yes	RR	7.8%	16.6%	Multiple prior images reduced RR and increased CDR and PPV of recall	Age
						PPV of recall	5.6%	NR		
						CDR	Single prior (4.3/1000) Multiple prior (6.6/1000)	N/A		
Frankel et al.1995^18^	USA	Retrospective study	DM: *n* = 3386	Not reported	Yes	AIR	3%	7%	Substantially fewer abnormal screening interpretations were made when mammography had been performed previously and when the prior films were available for comparison.	None
						Number of cancers detected	41%	32%		
Burnside et al. 2002^31^	USA	Retrospective study	FS: *n* = 48,281 screening n=: 9825 Diagnostic: *n* = 38,456	Radiologists (numbers not reported	Not reported	RRs	Screening: 3.8%	4.9%	Screening: prior images reduced recall rates but not biopsy rate recommendation and cancer detection. Diagnostic: previous mammograms increased true-positive findings	None
						CDR	Screening: 5.2/1000 Diagnostic: 39/1000	5.5/100011/1000		
						Biopsy performed BRR	Screening 1.2% Diagnostic: 7.6% Diagnostic 9.4%	1.4%3.0%4.3%		
						Biopsy yield	Screening: 44% Diagnostic: 51%	40%38%		
Sumkin et al. 2003^22^	USA	Observer performance study	FS: *n* = 128	Radiologists (*n* = 12)	Yes	Accuracy	72%	65%	Availability of prior images improved accuracy and specificity without affecting sensitivity.	None
						Sensitivity	71%	73%		
						Specificity	72%	63%		
Yankaskas et al. 2011^34^	USA	Retrospective study	Unclear if they are DM or FS: *n* = 1157 980	Radiologists (number not reported)	Yes	RR	6.9%	14.9%	Comparison mammograms: reduced CDR, RR, sensitivity, and PPV, but increased specificity,	Breast density
						Sensitivity	78.9%	87.4%		
						Specificity	93.5%	85.7%		
						CDR	3.7%	7.1%		
						PPV	5.4%	4.8%		
Hakim et al. 2015^20^	USA	Observer performance	DM+DBT 153 (50 cancers, 60 normal, 43 benign	Radiologists (*n* = 8)	Yes	RRs	Cancer: 81% Non-cancer: 33%	85%50%	Prior images reduced RR in cancer and non-cancer images but decreased sensitivity. Prior images had no effect on ROC AUCs and specificity.	None
						AUC	83%	83%		
						Sensitivity	7% reduction	NR		
						Specificity	Reported *p*-values only	Reported *p*-values only		
Roelofs et al. 2007^26^	Netherlands	Observer performance	Digitised FS: *n* = 160	Radiologists (*n* = 12)	Yes	LROC/LLFS	26%	19%	Prior mammograms increased Lesion localisation.	None
Thurfjell et al. 2000^28^	Denmark	Observer performance	FS: *n* = 150 (SDC: 49; IC:12; normal:89	Radiologists (*n* = 3)	Yes	FPR	3.7%	13.3%	Prior images increased specificity, reduced FPR with no change in sensitivity	None
						Sensitivity	62%	69%		
						Specificity	96%	87%		
Taylor-Phillips et al. 2012^27^	UK	Observer performance	DM & FS: *n* = 160 (cancer:66)	Radiologists (*n* = 4) Radiographers (*n* = 4)	Yes	JAFROC	87%	83%	Prior images improved JAFROC and reduced RR and FPR	None
						FPR	40%	59%		
						FPR RR JAFROC FOM difference	36% 43% 0.3%	49%53%Baseline		
Varela et al. 2005^29^	Netherlands	Observer performance	FS: *n* = 198	Radiologists (*n* = 6)	Yes	AUC	79.6%	76%	Prior images improved performance	None
Soh et al. 2014^21^	Australia	Observer performance	DM: *n* = 200 (screening+diagnostic)	Radiologists (*n* = 10)	Yes	ROI FOMs	88%	85 %	Prior images had no effect on ROI FOMs, sensitivity, and specificity	Reading location
						Sensitivity	80%	76%		
						Specificity	91%	87%		
Trieu et al. 2023^30^	Australia	Retrospective study	Screening (*n* = 540; Cancer:179; Normal: 361	Radiologists (*n* = 612)	Yes	ROC AUC	0.782–0.820	0.814	AUC and Sensitivity without priors were higher, but specificity decreased without prior images. RRs were only lower if the priors were obtained with the same vendor technology as the current images	Breast density and technology
						Sensitivity	0.712–0.785	0.803		
						Specificity	0.771–0.787	0.749		
						RR	0.353–0.461	0.444		

AIR: abnormal interpretation rate; AUC: area under ROC; BRR: biopsy recommendation rate;CDR: cancer detection rate; FPR: false-positive rate;IC, interval cancer; JAFROC: jacknife free-response receiver operating characteristic; LLFS: lesion localised fraction score; LROC: location ROC; NR: values not reported;; ROC: receiver operating curve; RR: recall rate;SDC, screen-detected cancers.

a Figures estimated from ROC AUC curves.

### Critical appraisal within sources of evidence

Appendix 1 summarises the strengths and weaknesses of the studies reviewed according to the STARD guidelines. 10 of the studies reviewed were prospective^17,19–22,25–29^ and 5 were retrospective analyses.^18,30,31,33,34^ All studies provided the aim, described the reference standard, blinded readers, and described the flow and timing of the index test. 10 studies described the participants,^17,19–22,26–30^ 3 studies provided limited participant information^31,33,34^ and only 2 studies did not provide information about participants’ demographics.^18^ Almost all of the studies (14/15) provided information about the index test; however, the retrospective studies did not establish the reference standard prior to the index test. Only one study described how the readers were sampled^21^ or assessed the reproducibility of the findings.^20^ Information about the expertise of the reader were reported in 11 studies.^17,19–22,25–28,33,34^ All studies provided information about the methods for calculating reader performance and uncertainty. The characteristics of the females whose mammograms were used (such as ethnicity, age) were clearly described in 10 studies, but this information was unclear in the remaining 5 studies. 13 of the studies described disease distribution and severity.

Appendix.

Overall, most of the studies were well conducted in terms of the reference standard, blinding of readers, index text, methods for calculating or comparing measures of diagnostic accuracy, and the statistical methods used to quantify uncertainty. Weaknesses were noted in terms of reproducibility, description of the expertise of the readers, and the characteristics of the population studied. Only seven of the studies reported the interval between the prior and current mammograms, which ranged from 9 months to 4.5 years. Many of the studies (11/15) did not adjust for any confounding factor, two studies adjusted for breast density and age, and one study adjusted for reading location (Table 2).

### Results of individual sources of evidence

Table 2 summarises the characteristics and results of studies reviewed. The results of studies comparing reader performance with and without prior images can be mapped into two themes: (1) diagnostic performance evaluation; (2) RRs.

#### Diagnostic performance evaluation

Of the three studies that assessed CDR, only two of these studies assessed CDR per 1000 women screened and the reported values ranged from 3.7 to 5.2% with prior mammograms and 5.5 to 7.1% without prior mammograms. One of the studies reported that the availability of prior mammograms did not affect the CDR,^31^ one reported an increase in CDR,^33^ and one study reported a decrease in the CDR.^34^ Overall, evidence for the association between the availability of prior mammograms and CDR is limited and unclear.

Among the five studies that compared performance using ROC AUC, three reported ROC values ranging from 79.6 to 83% (Mean: 80.16%) with prior mammogram availability and 76 to 83% (Mean: 80.68%) without prior images available. Four of the five studies showed that there is no difference in diagnostic accuracy between reading with and without prior mammograms,^17,20,25,29,30^ and one study showed that when analysis was stratified by breast density, AUC values without prior images were significantly higher than AUC obtained when prior and current mammograms were acquired using the same vendor technology.^30^ JAFROC and LROC, which rely on the reader’s ability to localise the lesion within an acceptable radius, were each assessed in one study and were found to improve with the availability of prior mammograms.^26,27^

Six out of seven studies reported sensitivity values ranging from 62 to 86% (mean = 73.3%) with prior mammograms and 69.4 to 87.4% (mean = 75.8%) without prior mammograms, and one study reported a 7% reduction in sensitivity with prior mammograms without providing the sensitivity values obtained. Many of these studies (5/7) indicated that availability of prior mammograms did not have any effect on reader sensitivity.^21,22,25,28^ The studies that used specificity reported values ranging from 72 to 96% (mean = 87.5%) with prior mammograms and 63 to 87% (mean = 80.5%) without prior mammograms. Most of the studies (5/7) indicate that availability of prior mammograms increased specificity.^22,25,28,30,34^

Reported FPRs ranged from 3.7 to 36% (mean = 19.9%) with prior mammograms and 13.3 to 49% (mean = 31.4%) without prior mammograms. Data from the studies show that prior mammograms reduced FPR.^27,28^ The only study that assessed abnormal interpretation rate reported that the availability of prior mammograms reduced AIR by 4%.^18^ The two studies that examined the impact of prior mammograms using PPV showed significant improvement.^25,34^

#### Recall rates

The reporting of RRs is included here as the rate of screening females recalled for follow-up imaging test due to suspicion of cancer requiring further imaging and biopsy is closely linked and reported as separate entities. While there is a relationship between specificity and RRs in screening mammography, RRs are also linked to sensitivity and TP identification and have been the discrete focus of some studies included in this review.

Five of the six studies that examined RRs showed that the availability of prior mammograms reduced RRs. RRs with prior mammograms ranged from 3.8 to 57% (mean = 26.6%), which was significantly less that that reported without prior mammograms [4.9% to 67.5% (mean = 37.9%)].^19,20,30,31,33^ There was limited literature on the association between access to prior mammograms and PPV of recall.^33^

## Discussion

The findings suggest that the availability of prior mammograms improve specificity and PPV and reduce FPRs and RRs. In the studies identified through our search, access to prior mammograms did not improve reader sensitivity, CDR, and AUC values. Limited data were available to establish the effect of prior mammographic availability on PPV of recall.

The findings above can be explained by a few factors. Breast cancer presents with an array of features including masses, stellate lesions, non-specific densities, and architectural distortions.^16^ While some of these features may be easy to identify, other features such as architectural distortions and non-specific densities may be subtle and associated with benign conditions or are indeterminate.^35^ The detection and discrimination of these subtle and indeterminate lesions may be challenging to radiologists regardless of prior mammogram availability,^36^ if lesions are not visible in prior mammograms and do not elicit significant changes in current mammograms.^36^ Also, even if a lesion is apparent in the prior and current mammograms, but comparison with prior mammograms did not show significant changes in the lesion, this radiological feature may be dismissed. Therefore, comparison with prior mammograms may only result in the interpretation that a lesion is malignant when significant changes are observed in the lesion detected in current images. Thus, it is not surprising that diagnostic performance metrics such as sensitivity and ROC AUC, which depend on readers’ ability to distinguish between normal and abnormal image features, and CDR, which depend on the ability of the readers to identify and classify lesions in mammograms, do not change in the presence or absence of prior mammograms.^21,22,25,28^

Conversely, in the absence of significant differences in the radiographic features of current and prior mammograms or changes in lesion appearance between these two sets of mammograms, the radiologists’ interpretation that the female has no cancer signs may increase. Such interpretation differences may explain the increased specificity and lower false-positive and abnormal interpretation rates when prior mammograms are available. This in turn affects the recall rate, as improved specificity should lead to reduced recall rates if false-positives are improved simultaneously.

Although screening mammograms are performed on asymptomatic females, prior mammograms can be considered as a form of imaging history akin to written clinical notes about a person’s medical history, which may influence the pre-test probability of breast cancer, particularly if these mammograms show features suggestive of cancer. Evidence in the literature shows that embedded imaging history has a positive influence on radiologists’ decisions not to recommend further assessment by reducing the number of females recalled at screening.^19,20,31,33^ In Australia, females recalled at screening undergo any of, or a combination of, mammography spot and compression views, ultrasound, and DBT assessment as well as biopsy.^37^ Only 1–2% of females recalled at screening return a positive result after these series of testing, and as such, increased multiple testing can increase psychosocial harms and may deter some females from rescreening.^38^ The number of females recalled at screening is used as one of the most important indicators of the performance of a breast cancer screening program.^39^ Interestingly, the literature reports that the availability of prior mammograms reduced RRs and improved the PPV of recall, suggesting that prior mammograms have a positive impact on recall decisions and the females recalled benefitted from these further assessments.

Whilst the findings of the review question the role of prior mammograms in improving cancer detection, particularly in small lesions which is one of the goals of screening mammography, it supports the use of prior mammograms to reduce the number of females recalled to assessment clinics. Low FPRs and low RRs that do not affect sensitivity are also important to a screening program because they improve workflow, reduce cost and harms such as patient anxiety, overdiagnosis, and overtreatment. The availability of prior mammograms reduces RRs and FPRs and increases specificity. Surprisingly, reading mammograms with the prior images did not improve sensitivity and CDRs. While these findings suggest that prior mammograms can be considered an important strategy to improve the specificity and RRs of screening programs and reducing radiologists’ assessment workload, questions will remain about the high visual workload in reviewing prior images in the screening round.

Whilst the current review has identified 15 studies that assessed the impact of prior mammograms on diagnostic efficacy, it is important to note that most of these studies focused on different performance metrics. Sensitivity and specificity were examined in only seven studies, with other metrics considered in four or fewer studies. The differences in performance metrics across studies, wide variations in the study methodologies (retrospective analysis and experimental observer performance study), sample sizes, imaging technologies, and participant characteristics limits the comparison of results across the studies reviewed. For example, retrospective studies mostly did not report the number and experience of radiologists that interpreted the mammograms and almost all the studies prior to 2011 were based on the older FS technology, with a few of these combining FS, FFDM and DBT technologies. The Digital Mammographic Imaging Screening Trial (DMIST) which compared FS and FFDM has shown that FFDM performed better than FS mammography in females with dense breasts. Also, only one of the studies in this scoping review that assessed reader performance adjusted for the effect of breast density, but did not report the results of this adjustment; hence it could be impractical to compare results of studies that use a blended technology.^40^

Other important factors that should be considered when interpreting the results of published studies include adjustments for factors that confound reader performance such as breast density, characteristics of the population and lesions included in the data set, and characteristics of the radiologists that interpreted the images. For example, 12 of the studies did not adjust for any confounding factor, and only 2 studies accounted for breast density and age, which are established factors limiting cancer detection in FFDM. In addition, even though the distribution of cancer were reported in many studies, the characteristics of the lesions included in the data sets were not described as required by the STARD guidelines.^24^ Furthermore, the small numbers of readers in the published studies and lack of information on their sampling strategy limit the generalisability of the results.

The methods of assessing performance across most of the studies reviewed focused on case-based analyses which is appropriate to a screening scenario. Only two studies performed lesion-level analyses using JAFROC and LROC,^26,27^ with the results of these studies insufficient to draw definite conclusions about the impact of prior mammograms on lesion sensitivity performance, although we recognise that lesion sensitivity is not generally associated with the performance of screening programs. In addition, the imaging features of cancer, such as asymmetric densities, generally require prior mammograms for identification but these were not accounted for in statistical analysis in published studies. Finally, only 46% of the studies reported the intervals between the prior and current mammograms which demonstrated wide variation and, in some cases, very short intervals between prior and current mammograms (9 months to 3 years) in published studies.^19,20,22,25,27,34^ Only one study included prior mammograms obtained 3–4.5 years before the current mammograms.^28^ These short intervals and variations across studies may potentially bias the outcomes of published studies. This is because slow growing cancerous lesions generally require an interval of 2–5 years to show subtle changes on mammograms and shorter intervals may limit radiologists’ ability to detect subtle changes related to breast cancer, noting that national programs such as BreastScreen Australia have a recommended interval of 24 months.

The limitations of the studies reviewed highlight the need for well-designed studies that consider current technologies, assess performance at case and lesion levels, and account for all the factors that confound radiological diagnosis. Such studies may provide a better understanding of the relevance of prior mammograms in breast cancer screening programs and how their role is validated in radiologists’ education for breast cancer early detection.

This scoping review has a few limitations. Firstly, the literature search was conducted in the English language and only studies published in the English language were included in the review. Secondly, many retrospective studies that used the statistics from screening populations and did not determine the index test prior to the interpretation or provide information of the radiologists that interpreted the index test were included. To our knowledge, this scoping paper is the first to review the literature on the availability of prior mammograms and diagnostic efficacy. The outcomes of this review should inform future studies to address the deficiencies in the literature and provide informed evidence for the use or prior mammograms in the clinical setting. The review of prior images has somewhat been unchallenged in radiology, always anecdotally considered a positive task; however, the evidence could be clearer.

## Conclusion

Evidence show that access to prior mammograms reduces RRs, FPRs, abnormal interpretation rates, and increased specificity, however this does not show an improvement in sensitivity and the CDR. Future studies that consider reader performance at case and lesion levels and account for factors that confound radiological diagnosis are needed to confirm the true effect of prior mammograms on performance in the digital era.
