# Supplementary material for: Does the availability of prior mammograms improve radiologists’ observer performance?—a scoping review
Source: BJR Open. 2023 Oct 18;5(1):20230038. doi: 10.1259/bjro.20230038 (PMC10630973; doi:10.1259/bjro.20230038)
Supplement: Supplementary file 1 — Appendix. [file bjro.20230038.suppl-01.docx]

Appendix 1: Critical appraisal of the articles identified

| Author | Aim | Participant | Reference | Blinding | Index | Flow & timing | Participant Sampling | Reader expertise | Data collection | Methods for calculating performance and uncertainty | Characteristics of the Population studied | Disease distribution and severity | Reproducibility |
| --- | --- | --- | --- | --- | --- | --- | --- | --- | --- | --- | --- | --- | --- |
|  |  |  |  |  |  |  |  |  |  |  |  |  |  |
| Callaway et al. (17) | Yes | Yes | Yes | Yes | Yes | Yes | Not described | Yes | Prospective | Yes | Yes | Yes | No |
| Hakim et al. (19) | Yes | Yes | Yes | Yes | Yes | Yes | Not described | Yes | Prospective | Yes | Yes | Yes | No |
| Kim et al.  (25) | Yes | Yes | Yes | Yes | Yes | Yes | Not described | Yes | Prospective | Yes | Yes | Yes | No |
| Hayward et al. (33) | Yes | Partial. Number not reported | Yes | Yes | Yes | Yes | No | No | Retrospective study | Yes | Yes | Yes | No |
| Frankel et al. (18) | Yes | No | Yes | Yes | Yes | Yes | No | No | Retrospective study | Yes | Yes | Yes | No |
| Burnside et al. (31) | Yes | Partial. Number not reported | Yes | Yes | Yes | Yes | No | No | Retrospective study | Yes | No | No | No |
| Sumkin et al. (22) | Yes | Yes | Yes | Yes | Yes | Yes | Not described | Yes | Prospective | Yes | Partial | Yes | No |
| Yankaskas et al. (34) | Yes | Partial. Number not reported | Yes | Yes | Yes | Yes | Not described | Yes | Retrospective study | Yes | Yes | Yes | No |
| Hakim et al. (20) | Yes | Yes | Yes | Yes | Yes | Yes | Not described | Yes | Prospective | Yes | Yes | Yes | Yes |
| Roelofs et al. (26) | Yes | Yes | Yes | Yes | Yes | Yes | Not described | Yes | Prospective | Yes | Yes | Yes | No |
| Thurfjell et al. (28) | Yes | Yes | Yes | Yes | Yes | Yes | Not described | Yes | Prospective | Yes | Unclear | Yes | No |
| Taylor-phillips et al. (27) | Yes | Yes | Yes | Yes | Yes | Yes | Not described | Yes | Prospective | Yes | No | Yes | No |
| Varela et al. (29) | Yes | Yes | Yes | Yes | Yes | Yes | Not described | No | Prospective | Yes | Yes | Yes | No |
| Soh et al. (21) | Yes | Yes | Yes | Yes | Yes | Yes | Yes | Yes | Prospective | Yes | No | Yes | No |
| Trieu et al.(30) | Yes | Yes | Yes | Yes | Yes | Yes | No | Yes | Retrospective study | Yes | No | Yes | No |
